# Pre-target neural oscillations predict variability in the detection of small pitch changes

**DOI:** 10.1371/journal.pone.0177836

**Published:** 2017-05-18

**Authors:** Esther Florin, Dominique Vuvan, Isabelle Peretz, Sylvain Baillet

**Affiliations:** 1 McConnell Brain Imaging Center, Montreal Neurological Institute, McGill University, Montreal, Quebec, Canada; 2 Institute of Clinical Neuroscience and Medical Psychology, Medical Faculty, Heinrich-Heine University Düsseldorf, Düsseldorf, Germany; 3 Centre for Research on Brain, Language and Music, Montreal, Quebec, Canada; 4 Psychology Department, Skidmore College, Saratoga Springs, NY, United States of America; 5 Department of Psychology, International Laboratory of Brain, Music, and Sound Research, University of Montreal, Montreal, Quebec, Canada; Australian Research Council Centre of Excellence in Cognition and its Disorders, AUSTRALIA

## Abstract

Pitch discrimination is important for language or music processing. Previous studies indicate that auditory perception depends on pre-target neural activity. However, so far the pre-target electrophysiological conditions which enable the detection of small pitch changes are not well studied, but might yield important insights into pitch-processing. We used magnetoencephalography (MEG) source imaging to reveal the pre-target effects of successful auditory detection of small pitch deviations from a sequence of standard tones. Participants heard a sequence of four pure tones and had to determine whether the last target tone was different or identical to the first three standard sounds. We found that successful pitch change detection could be predicted from the amplitude of theta (4–8 Hz) oscillatory activity in the right inferior frontal gyrus (IFG) as well as beta (12–30 Hz) oscillatory activity in the right auditory cortex. These findings confirm and extend evidence for the involvement of theta as well as beta-band activity in auditory perception.

## Introduction

The ability to discriminate pitches underlies a number of important cognitive functions. It is important for auditory scene analysis, where it facilitates stream segregation in the service of auditory object formation [1]. For language processing, the pitch content of speech signals conveys important lexical, syntactic, and semantic information [2]. In the domain of music, the ability to detect small differences between pitches is necessary for the hierarchical organization of pitch known as key or tonality [3]. Therefore, unpacking the neural underpinnings of this seemingly basic ability of pitch discrimination has far-reaching consequences for the understanding of the auditory system and cognition.

Early electrophysiological investigations into pitch discrimination used an auditory oddball task to determine brain responses to changes in pitch frequency [4, 5]. Two responses are typically evoked by auditory oddball targets: the mismatch negativity (MMN) and the P3. Interestingly with magnetoencephalography (MEG) the neural generators of MMN have been localized in the inferior frontal, the superior temporal as well as the orbitofrontal cortex [6]. For melodic deviants this network is more pronounced in the right hemisphere [7].

Investigations in both vision and audition suggest that perception critically depends on the amplitude (power) and phase of pre-target neural activity [8–17]. In the auditory modality, delta and theta oscillations are important for temporal integration and the parsing of speech [18–20] as well as the detection of sounds [14]. Moreover, delta and theta frequencies have been determined as potential entrainment frequencies so that their phase indicates fluctuations in attention [21, 22]. Delta and theta oscillations were suggested to be correlated with attentional fluctuations [23]. Hence, as proposed by the auditory dynamic attention theory [24, 25], they might play a crucial role in pitch discrimination in regularly spaced tone sequences.

As a marker of regional neural excitability, alpha activity is also considered to be related to attentional processes [16]. Performance in beat perception and temporal expectancy is particularly modulated by beta activity [26, 27]. Therefore auditory beta activity could be related to motor preparation and the top-down influence of the motor system on the auditory cortex. The gamma band has been associated with auditory feature binding and the matching of acoustical cues to representations in memory [28, 29].

Studies of pre-target activity have focused on the detection of a target stimulus, whether that be a tone or a gap within a sound in the auditory domain [9, 14, 30–32] or a flash of light or target shape in the visual domain [8, 12, 33]. Studies using detection paradigms tend to find effects of oscillatory phase on perception. Specifically, phase can be construed as playing an inhibitory role on perception, such that a stimulus will not be perceived if the phase of the accompanying brain activity is not entrained to its onset. Supporting this idea two recent studies could demonstrate with 4Hz transcranial alternating current stimulation that phase entrainment accelerates the perception of target sounds [34, 35].

Interestingly, a study that has employed a visual discrimination task [15] found effects of oscillatory power on perception. Discrimination requires further processing past simple detection (i.e. comparison of luminance). Therefore the power of neural oscillations may reflect the strength of the perceptual signal that reaches higher-level processing stages.

Based on these findings we hypothesized that pre-target oscillatory activity in the brain areas known to be engaged in pitch discrimination can predict the correct processing of near-threshold auditory stimuli. To test our hypothesis, we used a simple pitch discrimination task in which participants were required to indicate within a sequence of 4 tones whether the last tone was the same or different. This task has been extensively studied with EEG [36, 37].

The second hypothesis of our study was that functional connectivity between the auditory cortex (AC) and the inferior frontal gyrus (IFG) plays a crucial role for the detection of near-threshold auditory stimuli. This hypothesis is based on the finding that the activity of the right planum temporale is linearly modulated by increases in the magnitude of a pitch change [38]. In particular, the functional and anatomical connections between the auditory cortex and inferior frontal gyrus are important for pitch discrimination [39–44]. Due to the limited temporal resolution and indirect measurement of neural activity in those functional and diffusion-weighted MRI studies, the dynamic neuronal brain processes that underlie pitch discrimination have not been studied yet. Therefore, the electrophysiological mechanisms that precede, and enable successful pitch discrimination remain poorly understood.

To study these two hypotheses, we analyzed the neural activity and the functional connectivity within the AC-IFG network during the detection of small pitch changes. We used MEG source imaging [45], because it provides a high temporal resolution combined with a spatial resolution that previous EEG studies lacked.

## Materials and methods

### Participants

19 healthy participants were recruited for this study (20–44 years; median: 26 years; 9 female; education: median 18 years (12–23 years); formal musical training: median 1 year (0–13 years)). All participants were right-handed (self-report) and had normal hearing in the frequency range of our stimuli (pure-tone audiometry). The study was approved by the Montreal Neurological Institute’s ethics committee (NEU 12-023), in accordance with the Declaration of Helsinki. All participants gave written informed consent and were compensated for their participation.

### Stimuli

Participants were presented with a 2-second sequence consisting of four pure tones, i.e. one tone was presented every 500 ms. Each tone was 125 ms in duration, with 10 ms on- and off-ramps. The first three tones were always pitched at A4 (440 Hz). In half of the trials, the last tone was identical to the previous three tones (standard trials). In the other half of the trials, the last tone presented had a different pitch than the first three (deviant trials). The deviant trials were manipulated in a fully-crossed design according to two factors: the size and the direction of the pitch change. The pitch difference was either i) two semitones (easy condition) or ii) 6.25% of a semitone (hard condition) with respect to the first three tones in the sequence. Half of the presented pitch differences were upwards compared to the standard tone and the other half downwards. Two semitones are easily detectable by individuals with normal hearing, whereas 6.25% of a semitone is at the threshold for pitch change detection [1, 46]. We chose to keep the deviant tone the same for all participants, rather than adjusting the pitch difference according to the individual participant’s ability. Thereby we obtained a wide variety of behavioral performances. This design allowed us to relate behavioral differences to the neural activity of each individual. The performance to detect the hard deviant varied from 0–98% across all subjects (see S1 Table), implying that the broadest range of possible performances was covered. This on the other hand also implies that for 8 subjects the level of performance was lower than 50%.

### Experimental procedure

Participants received both oral and written instructions that on each trial, they would hear a series of four notes, and asking them to make speeded judgments by pressing a button with the index finger. Ten of the participants were instructed to respond to a deviant tone with a button press using the left index finger, and for the standard tone using the right index finger. The response pattern was the opposite for the other nine subjects. This procedure was chosen so that neural activity due to the motor response would be balanced bilaterally in order to minimize its influence on possible lateralization effects involved in pitch discrimination. Participants were instructed to answer according to their best knowledge and were not informed that there were different types of pitch changes. We chose this approach to reduce a potential response bias in particular for subjects who did not detect the hard deviant tones.

A constant inter-trial interval duration (2 s) was used during which participants were required to make their judgment. Although this design made the start of the next trial predictable, it was still impossible for participants to predict which condition was ahead. The constant inter-trial interval ensured that early differences in oscillatory activity during the first three tones were not due to an unexpected start of the trial. Participants were asked to fixate on a cross centered on a projection screen placed at a comfortable distance in front of them in order to minimize eye movements. The audiovisual stimuli were presented using E-Prime 2.0 (Psychology Software Tools, Inc., Sharpsburg, PA, USA). Sounds were presented binaurally using non-magnetic MEG-compatible earphones (E-A-RTONE 3A, Aearo Technologies, Indianapolis, USA). The intensity level of the sound presentation was adjusted to a comfortable level for every participant. Responses were recorded using two Lumitouch key pads (Photon Control, Burnaby, BC, Canada).

Each participant was presented with a total of 600 stimulus sequences (trials), of which 300 were of the standard type, and 150 each were of the easy and hard types. The presentation of the different trial types was randomized. Participants performed 80 trials per acquisition block, except the last acquisition block, which consisted of only 40 trials. Between each block, the participants were given a break of self-determined length. The participants did not receive feedback on the accuracy of their responses.

### Data acquisition

The participants were measured in a seated position using a 275-channel VSM/CTF MEG system with a sampling rate of 2400 Hz (no high-pass filter, 660 Hz anti-aliasing online low-pass filter). Magnetic shielding was provided by a magnetically-shielded room (MSR) manufactured by NKP (NKK Plant Engineering Corporation, Yokohama, Japan) with 3-layer passive shielding. Before the actual recording, the participants were tested for possible magnetic artifacts in a rapid preliminary MEG run. Participant preparation consisted of affixing 3 head-positioning coils to the nasion and both pre-auricular points. The positions of the coils were measured relatively to the participants’s head using a 3-D digitizer system (Polhemus Isotrack, Colchester, USA). To facilitate anatomic registration with MRI, approximately 100 additional scalp points were also digitized.

A T1-weighted MRI of the brain (1.5 T, 240 × 240 mm field of view, 1 mm isotropic, sagittal orientation) was obtained from each participant either at least one month before the MEG session or after the session. In case the MRI was obtained before the MEG, a waiting period between MRI and MEG recordings was adhered to in order to prevent potential magnetic contamination due to the increase of “magnetic noise” from the participant after the MRI acquisition [47]. For subsequent cortically-constrained MEG source imaging, the nasion and the left and right pre-auricular point were first marked manually in each participant’s MRI volume. These were used as an initial starting point for registration of the MEG activity to the structural T1 image. An iterative closest point rigid-body registration method implemented in Brainstorm [48] improved the anatomical alignment using the additional scalp points. The registration was visually checked and improved manually, if necessary.

Electrocardiography (ECG), electrooculography (EOG), and EEG were recorded using non-magnetic MEG-compatible electrodes. ECG was captured using a pair of electrodes placed across the participant’s chest (one above the inferior extremity of the left rib cage and one over the right clavicle). Similarly, a second pair of electrodes was attached above and below one eye to detect eye-blinks and large saccades (EOG). Finally, we recorded EEG from two standard electrode locations: CZ and PZ. The EEG reference was placed on the right mastoid. These additional recording channels were all sampled synchronously with the MEG signals (2400 Hz).

At the beginning of each MEG recording block, the location of the participant’s head within the MEG helmet was measured by energizing the head-positioning coils, following standard procedures. A 2-min empty-room recording (no person in the MSR) with the same acquisition parameters as during task performance was obtained before the experiment started. This recording was used to estimate sensor and environmental noise statistics for subsequent MEG source modeling, as detailed below.

### Data pre-processing

To minimize contamination from environmental noise, the MEG data were corrected using the manufacturer’s 3rd-order gradient compensation system (no parameter setting required). After recording, all the data were visually inspected to detect segments contaminated by head movements or remaining environmental noise sources, which were discarded from subsequent analysis. Across participants, an average 90% ± 7% of the trials were kept for the analysis.

Heart and eye movement/blink contaminations were attenuated by designing signal-space projections (SSP) from selected segments of data around each artifactual event [49]. We used Brainstorm’s default ECG and EOG detection processes and settings for the calculation of SSPs for this purpose [48]. The principal components that best captured the artifact’s sensor topography were manually selected as the dimension against which the data was orthogonally projected away from. In 13 of the participants, the first principal component was sufficient to attenuate eye blink artifacts, and for the other participants, the second component was also used. For heart beats, the artifact was sufficiently attenuated by a single SSP component in eight participants, with four of the participants requiring two components, and the remaining seven participants showing no visible contamination of the MEG traces due to heart beats. The projectors obtained for each participant were propagated to the corresponding MEG source imaging operator as explained below. Powerline contamination (main and harmonics) was reduced by complex match filtering with 1 Hz resolution bandwidth for sinusoidal removal, also available in Brainstorm.

The scalp and cortical surfaces were extracted from the MRI volume data. A surface triangulation was obtained using the Freesurfer segmentation pipeline, with default parameter settings, and was imported into Brainstorm. The individual high-resolution cortical surfaces (about 120,000 vertices) were down-sampled to about 15,000 triangle vertices (also with a Brainstorm process) to serve as image supports for MEG source imaging.

### MEG source imaging

The data was imported into Brainstorm using two distinct event-related epochs, with the fourth (target) tone being the event of interest. The first epoch window was defined to capture the oscillatory activity before the actual deviant tone was played: the raw data was extracted over [−2000, +1000]ms about the fourth tone presentation (time 0), with baseline over [−2000, −1600]ms. A second epoch window of [−100, +1000]ms around the fourth tone presentation was used for mapping the brain activity involved in pitch discrimination ([−100, 0]ms baseline). Baseline correction compensates for the DC drifts of MEG sensors. We could have used the longer time period in both cases and only changed the baseline. We opted for using different baselines, because with a common early baseline the evoked responses after the fourth tone would have been shifted in amplitude due to the different normalization based on the early baseline. Furthermore, the comparison to other auditory studies would be more difficult.

Forward modeling of neural magnetic fields was performed using the overlapping-sphere model implemented in Brainstorm [50]. MEG source imaging was obtained by linearly applying the weighted-minimum norm operator (Brainstorm, with default settings) onto the preprocessed data [45]. The weighted-minimum norm operator included an empirical estimate of the variance of the noise at each MEG sensor, as obtained from the empty-room recording described above. Note that the weighted-minimum norm operator is time-independent and therefore source estimation does not depend on epoch characteristics (baseline and duration).

### Analysis of the evoked components

The MMN and P3 components in the MEG and EEG data were identified by averaging trials according to their respective experimental condition (standard, hard, easy).

The N100 is a negative evoked response elicited by auditory stimuli and can be used as an index of attention [51, 52]. To determine attentional fluctuations, the amplitude of the N100 component was measured from CZ, following the presentation of the first tone for the hard-correct and the hard-incorrect trials, respectively. We assessed the N100 response to the first tone, rather than the subsequent tones, because the strongest evoked response is generated at the beginning of a pitch sequence.

We projected the difference between the standard condition and the hard or easy condition on the cortical surface as follows: we first filtered all source time series with a 20 Hz low-pass filter and took their absolute value. We then computed the difference between the processed source time series from the standard condition and the hard or easy condition, respectively. These activations were then normalized with a z-score based on the baseline from −100 to 0ms. Regions of interest (ROIs) in the right and left auditory cortices (AC) and inferior frontal gyrus (IFG) were then defined based on the strongest cortical MMN activation within these two cortical areas in the easy condition. We determined the ROIs on an individual level within 100ms–150ms after target tone presentation. For this purpose we used Brainstorm’s interactive user interface and extended the ROI over the anatomically-surrounding sources, which showed activation based on a z-score larger than 4.6. The details of the analysis of the evoked components are provided in the S1 Appendix.

### Analysis of the pre-target activity

We performed a time-frequency decomposition of MEG source time series extracted from each ROI using complex Morlet wavelets, as implemented in Brainstorm (mother wavelet with central frequency at 1 Hz and temporal resolution of 2 s at full width, half maximum). This resulted in time-frequency decompositions of the power of the source time series from the AC and IFG ROIs, over *t* = [−2000, +1000]*ms* for each frequency *f* = {4Hz, …, 30Hz}. These decompositions were then averaged across all elementary sources in each ROI per participant and per condition.

To compare the frequency components between conditions and to assess group-level significance within the time-frequency plane, we used the non-parametric cluster permutation approach of [53] and originally introduced by [54]. The first-level test-statistic was the t-value of a comparison between 2 conditions pooled across subjects. The t-test used was for equal variances. To further ensure similar signal-to-noise ratios, we determined for each subject the condition with the minimal number of included trials. Across all conditions we included a median of 44 trials (range 16–146) and for the hard condition comparison we included a median of 43 trial (range: 16–60). For all other conditions we then randomly drew the same number of trials from all available trials. This ensures that for each subject individually the same number of trials was chosen across conditions. The cluster statistic was defined based on the t-maps in the time-frequency plane from the comparison between conditions across subjects (sum of individual subjects’ t-values divided by square root of number of subjects (per time—frequency pixel)). This t-map was thresholded at a t-value of 2.1 and a minimal cluster size of 8 neighbours (*p* < 0.05, two-sided paired t-test). To test the significance of a cluster we performed 5000 randomizations as described in [53]. The significance of a cluster in the original data was determined for *p* < 0.05. To determine the significance within the time-frequency representation of one condition, the time-frequency maps were z-scored with respect to the baseline from [−2000, −1500]s prior to target onset. The z-values were then used for the cluster statistics, with a threshold of *z* > 2.1 and a minimal cluster size of 8 neighbours. The permutation was then performed in the same way as for the t-maps.

The functional connectivity between the IFG and AC regions was estimated with the corrected coherence value [55] at each available frequency bin, for each participant, and with respect to deviant task-correctness response {*hard* – *correct*, *hard* – *incorrect*}. Even though the corrected coherence measure accounts for the different number of trials in each condition, we additionally equalized the number of trials for each subject. To do so, we first determined the minimum number of trials from hard correct and hard incorrect trials median of 43 trial (range: 16–60). We then selected randomly the minimal amount of trials for the condition, which had more trials. Coherence was therefore calculated separately for all correct and incorrect detections. It was calculated using a time series constructed by concatenating the time window [−1500, 0]*ms* over all (in)correct trials of the respective condition. This time window includes the first three tones until the onset of the fourth tone. We then extracted the maximal corrected coherence between AC and IFG regions for each 2-Hz bin within 2–14Hz. The corrected coherence values were used to infer a possible linear relationship with subjects *d*′ through Pearson’s correlation coefficient. *d*′ was calculated for each individual participant based on performance [56]. In addition, the bias *c* was determined from the behavioral data. Further details are in S1 Appendix.

## Results

### Behavioral

**Fig 1:**
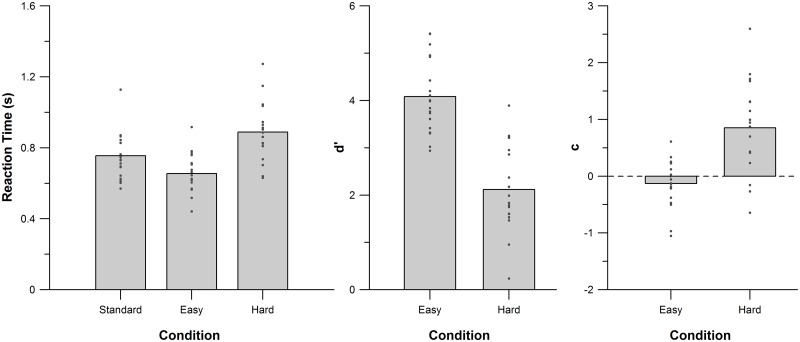
Behavioural data across conditions for reaction time, *d*′, and the bias (c).

Fig 1 shows reaction times (RT), *d*′, and *c* across the three conditions. The participants’ reaction times were submitted to repeated measures ANOVA with condition (standard, easy deviant, hard deviant) as a within-subjects variable. The effect of condition was significant (F(2, 36) = 31.23, MSE = .008, *p* < .001, $n_{p}^{2}$ = 0.63). In particular, RT was significantly faster for the easy deviant than for the hard deviant and standard tones (paired-sample t-test: hard vs. easy: t(18) = 7.97, *p* < .001; standard vs. easy: t(18) = 4.22, p = .003, Bonferroni-corrected for 3 comparisons). The reported times are with respect to the onset of the fourth tone until the button was pressed.

No participant had difficulty detecting the easy deviant tone (percent correct responses: 91.5%–100%). For the hard deviant tone condition, participants detected between 0% and 99% correctly (see S1 Table), implying that the broadest range of possible performances was covered for the hard deviant tones. Next, the participants’ signal detection scores were compared for the easy and hard deviant conditions. Both d’ and c of the easy and hard condition were calculated in relation to the standard condition. As expected, *d*′ scores were significantly higher for the easy than the hard deviant condition (t(18) = 7.78, *p* < .001), indicating greater discriminability between easy deviant and standard trials than between hard deviant and standard trials. Furthermore, all participants found the easy deviant trials to be highly discriminable, with all *d*′ values > 2. In contrast, the participants’ performances on the hard deviant trials were much more variable, ranging from nearly chance (*d*′ = .23) to very high (*d*′ = 3.89). Response bias *c* was significantly higher in the hard deviant than easy deviant condition (t(18) = 7.78, *p* < .001). This was driven by a strongly conservative bias (i.e., more likely to respond “no change”) in the hard deviant condition (*c* = .85), and a mildly liberal bias (i.e., more likely to respond “change”) in the easy deviant condition (c = −.13). Complete behavioural results can be found in S1 Table.

Finally, because the experimental session took about 1 hour inside the MEG, the possibility of fatigue affecting participants’ ability to do the task was assessed. The success rate (percent correct) showed no trend over runs, indicating that participant performance did not degrade over time (Friedman-test: *χ*^2^ = 6.6, *df* = 7, *p* = 0.48).

### Evoked responses

**Fig 2:**
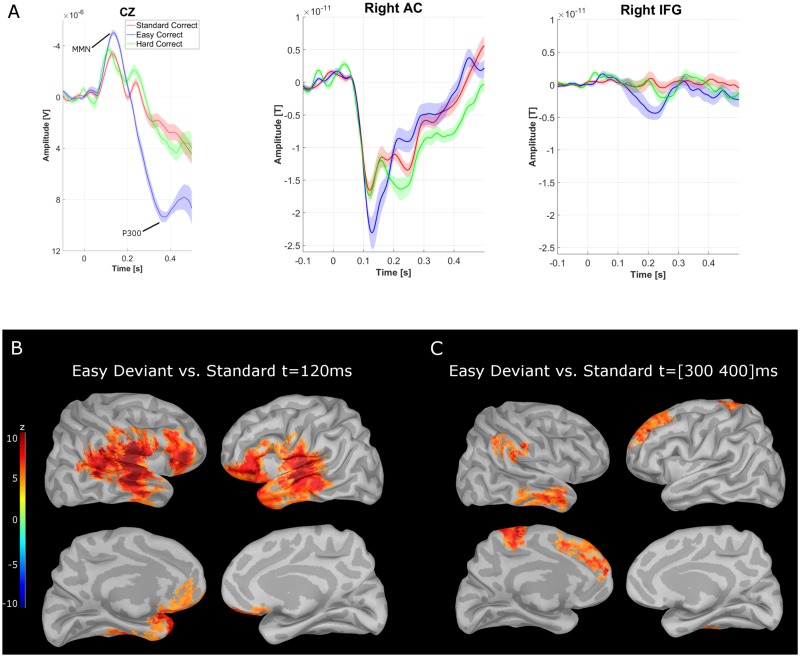
Evoked MEG response to the deviant tone. A: Averaged activity of the CZ EEG electrode, the Right AC and IFG: Note the MMN component and then the P300 component for the easy condition at CZ. The shaded areas in the ERP plots represent the standard error of the mean based on a within-subject design across the three conditions. B: Difference of source activity between the easy deviant tone and the standard tone averaged across all participants at time point 120 ms after stimulus presentation. C: Difference of the source activity between easy deviant tone and standard tone averaged over the time window from 300–400 ms after stimulus presentation. In panel B and C positive values indicate stronger activity in the deviant condition. All values are standardized with respect to the pre-target baseline from −100 to 0 ms. For the easy deviant tone there is a stronger activation in both auditory cortices, the inferior frontal gyrus, and the prefrontal cortex. The maps are thresholded for a z-value less than 4.6 (*p* < .05 Bonferroni corrected for multiple comparison). For the regions of interest the individual maps were used. They were then defined based on the strongest activation in each of the regions and as described in the methods.

Fig 2A shows the grand-averaged EEG trace on CZ. A MMN component is found at around 120ms, and a P300 component around 360ms. In the following, these two components are further analysed within the MEG data. In a first step, the MEG source activation for correct standard trials was subtracted from the source activation map corresponding to correct easy deviant trials. The resulting map was then normalized to z-values based on the baseline from −100 ms to 0 ms.

Fig 2B displays this activation map at the latency of the EEG MMN across participants from the CZ electrode. This difference map revealed the auditory cortices, the inferior frontal gyrus, and medial prefrontal regions to be more strongly activated for the easy deviant condition compared to the standard tone (*p* < .05, *z* < 4.6; Bonferroni corrected for 15,000 sources). The activation map within the auditory cortex showed a larger extent in the right hemisphere than in the left hemisphere (about 150 cm^2^ vs. 90cm^2^ of significantly activated surface areas), while the region activated within the IFG was larger for the left hemisphere than the right hemisphere (left: 68 cm^2^; right: 51 cm^2^). No significant differences in the MEG source activation maps were found between correctly-detected hard trials and standard trials. In the following analysis, the activation maps at the MMN occurrence were utilized to define regions of interest in the AC and IFG (see method section for details).

The map of cortical activity related to the P3 component was obtained by time-averaging each MEG source time series between 300ms and 400ms, to compensate for the variability in latency across participants. Interestingly, the difference map between easy and standard correct trials revealed significant activations within the right temporal lobe, right supra-marginal region, left motor area, and left medial frontal regions (*p* < .05, *z* < 4.6; Bonferroni corrected for 15,000 sources, see Fig 2C).

### Oscillatory neural activity within the auditory cortex and inferior frontal gyrus

**Fig 3:**
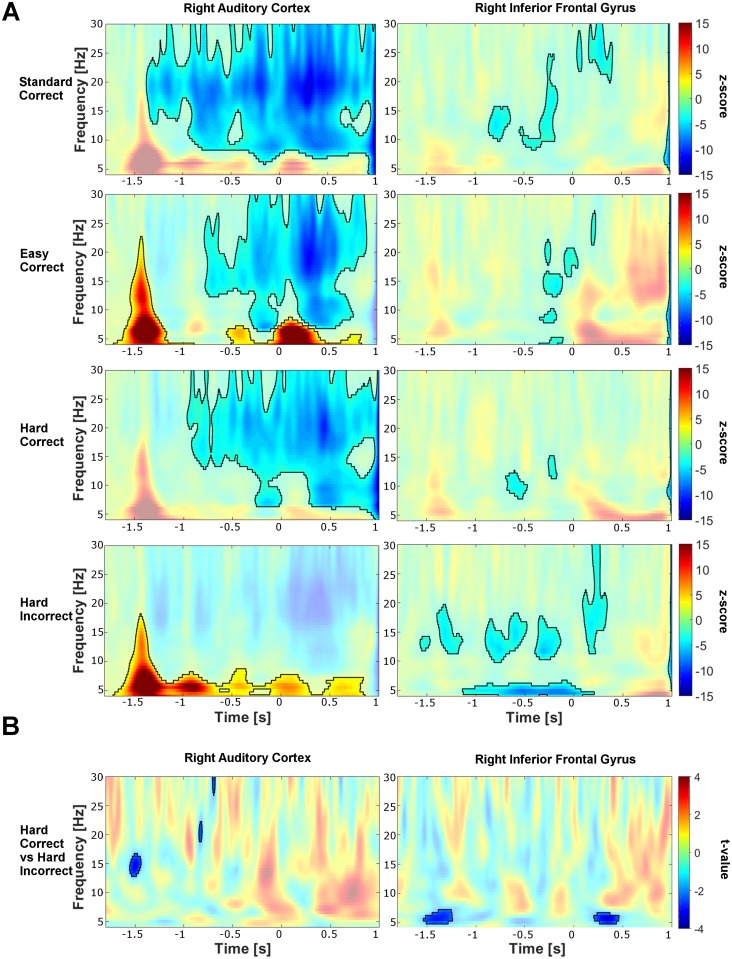
Time-frequency maps of the activity in the right auditory cortex and right inferior frontal gyrus. A: Time-frequency maps for the different conditions (standard tone, easy deviant, hard deviant). The first tone is played at −1.5 s and the target tone was played at 0 s. The time-frequency decomposition of the MEG source power was obtained with Morlet wavelets and the maps are cluster corrected for *p* < .05 as described in the methods section. The original z-maps were obtained by contrasting the activity from the baseline (−2 s to −1.5 s) to the task related activity. The significant areas in the time-frequency plane are enclosed by a black line; the non-significant changes are the surrounding transparent areas. B: Power difference between the correctly vs. incorrectly detected hard deviants. After the first tone is played (−1.5 s) lower power levels in oscillatory activity over the 5–8 Hz range are observed for correctly detected hard deviants. The maps are thresholded for *p* < .05, as described in the methods section.

**Fig 4:**
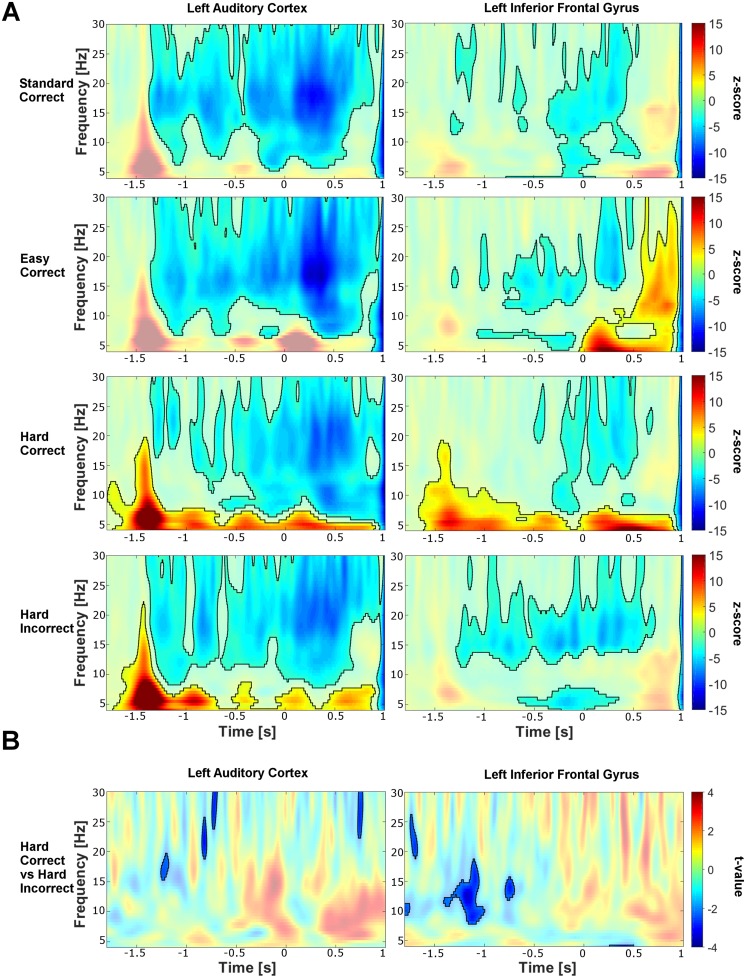
Time-frequency maps of the activity in the left auditory cortex and left inferior frontal gyrus. The same legend as for Fig 3 applies.

The time-frequency maps from the right AC and right IFG are shown in Fig 3 and for the left AC and IFG in Fig 4. Right after the onset of the first tone (time = −1500 ms), the amplitude of theta and alpha activity (4–12 Hz) increased. This effect was observed across all four conditions, but was not statistically significant for all conditions (cluster corrected across subjects, *p* < .05). However, we found that the lack of significance in the other conditions might be due to a cluster separation issue: In some of the conditions there is a connection between the pre-target cluster and the post-target increase. Therefore, the interpretation of the significant pre-target increase found in some of the conditions and its comparison to the other conditions remains ambiguous and needs to be interpreted with caution. Turning to the fourth target tone (time = 0 ms), we observed decreased activity in the alpha and beta band directly before the target tone was presented, with this effect being more prominent in AC than IFG (cluster corrected, *p* < .05). After the target tone was played, the decrease in both beta and alpha activity continued in particular in the left hemisphere (cluster corrected, *p* < .05).

To detect possible predictive components for correct or incorrect detection of hard deviant tones, we compared the spectrograms corresponding to the correctly and incorrectly detected hard trials. Comparisons were performed at the group level across the whole time-frequency plane, including the pre-target activity from [−2000, 0]*ms* and from 0.5 to 30 Hz (at a resolution of 0.5 Hz), with cluster correction for multiple comparisons. Before the target tone was played, i.e. before the participant knew whether the fourth tone was deviant or not, the power between 5 and 8 Hz (high theta range) in the right IFG and between 8–20 Hz in the left IFG was significantly lower for correctly vs. incorrectly detected trials (*p* < .05, permutation test; [53]). In the right and left AC significant pre-target power changes were detected in the beta range—with significant beta decreases after the first and second tone. Interestingly, the *post*-target power was significantly lower for correct than for incorrect trials in the right IFG from 5–8 Hz (*p* < .05); Fig 3b).

We also tested for a possible relation between the phase angle and the probability of correct detection of pitch changes in the hard condition following the analysis in [32]. Our analysis did not reveal any preferred phase in the theta or alpha frequency ranges (Friedmann-test across the detection probabilities for different phase angles). Interestingly, we did find a significant phase alignment effect at the presentation of the 4^*th*^ tone in the alpha range for the incorrectly detected trials in the left auditory cortices (AC l incorrect *α*: z = 4.74; p = 0.045 with Bonferroni correction for 16 comparisons).

To determine whether the results were driven by attentional fluctuations, we assessed the N100 response to the first tone in the sequence. The N100 is a standard response to a tone. Its temporal occurrence is similar to the MMN, which is found after a deviant tone. The N100 amplitudes for the hard-correct and the hard-incorrect trials were not significantly different (t(15) = 1.42, p = .18) and the correlation between *d*′ and the difference in N100 amplitude between trials with correct and incorrect responses was not significant (*ρ* = 0.04, p = .88).

### Inter-regional functional connectivity

**Fig 5:**
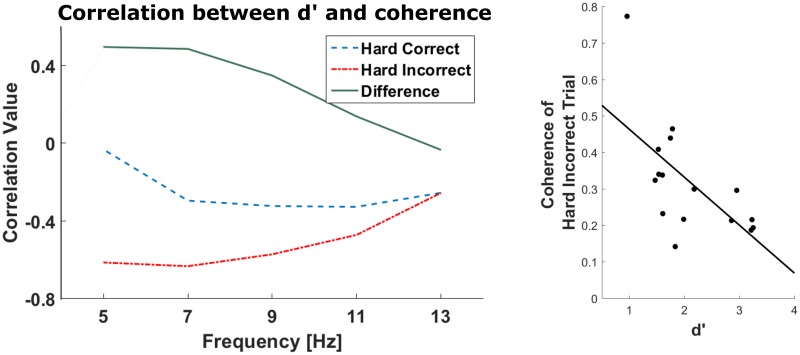
Correlation between coherence and *d*′. The correlation coefficient between the corrected coherence between right AC and IFG of each individual subject in 2 Hz steps with *d*′ is shown (i.e. Tick 5 Hz ≅ 4–6 Hz). The correlation between *d*′ and the coherence of the undetected hard deviants in the 6–8Hz range is the largest (*ρ*_*f*_ = −0.61). The individual values for this frequency range are shown on the right. Subjects with no/all correctly detected hard trials are not included in this calculation, because the difference cannot be calculated in these cases.

Previous studies have emphasized the role of anatomical and functional connections between the IFG and the AC for the correct processing of pitch changes [39, 43]. Our own results indicate differential oscillatory activity in AC and IFG between the correctly and incorrectly detected tones in the hard condition. In order to identify potential fluctuations in functional connectivity between IFG and AC, we obtained measures of coherence between the MEG source activity in these two regions during the presentation of the first three tones. We did find a difference in the averaged coherence for the hard incorrect trials between right IFG and right AC from 6–8 Hz (*ρ*_*f*_ = −0.61 p = 0.008 uncorrected, n.s. after Bonferroni correction for 15 comparisons; robust correlation *ρ* = −0.58, p = 0.0164, Fig 5), but no significant interaction on the left hemisphere.

## Discussion

We used MEG source imaging to analyze the time-resolved neural oscillatory activity and functional connectivity during pitch discrimination using a simple near-threshold pitch discrimination task [57].

Our results on pre-target activity show that the modulation of oscillatory activity from 5–20 Hz (theta through low beta band) in both IFGs were predictive of the successful detection of near-threshold pitch deviations. This is in agreement with the assumption that power of neural oscillations may reflect the strength of the perceptual signal that reaches higher-level processing stages. Additionally, we used fronto-temporal oscillatory coherence as a marker of functional connectivity. Here we did not find a clear result. Still, from 6–8 Hz there was a high correlation between coherence of hard incorrect trials and d’. This suggests that low-frequency fronto-temporal connectivity contributes to the accurate detection of subtle pitch changes in the healthy brain.

Additionally, and in agreement with previous neurophysiological work on *post*-target pitch discrimination [4, 5], both the MMN and P3 components were evoked in response to the pitch deviant. MEG source imaging of the MMN indicated that the AC and IFG were more strongly activated during the processing of deviant vs. standard tones, which is in line with previous fMRI and PET studies that have localized pitch processing in these regions (e.g. [38, 67]). For the P3 component we detected activations across the brain without a clear pattern. Previous MEG studies did not find significant differential activation for the P3 [58, 59]. This might be explained by the fact that the P3 is an entity originally defined based on EEG observations. It is generated in part by subcortical structures (i.e., thalamus, hippocampus, deep temporal regions; [60]) that are generally difficult to detect with MEG without resorting to an unusually large number of trial repetitions [61]. Our detected regions within the temporal lobe and the medial surface indicate that most likely deeper regions are involved, which we cannot localize further with the current study design.

One potential explanatory mechanism for our results is attentional modulation. Previous studies have related low-frequency phase information in the auditory cortex to changes in attention [21, 62]. To control for this potential effect of attentional drifts, we measured the N100 amplitude elicited by the first tone. The difference between the amplitude of the N100 in subsequently correctly vs. incorrectly detected deviants was not significant, and there was no significant correlation between the difference of the N100 amplitudes and *d*′. Furthermore, previous work [63] has indicated that alpha activity is related to the level of attention. In contrast, our findings are within the right IFG in the theta frequency range. Thus, at least based on the frequency content and the N100 amplitude, our results seem to not be mainly related to attentional fluctuations.

A second potential explanatory mechanism is temporal entrainment of the auditory cortex by stimulus presentation. The importance of oscillatory synchrony in the auditory system has been highlighted in two recent reviews [16, 18]. For speech processing in particular, oscillatory activity in the delta and theta range enables the auditory cortices to parse syllabic information when entrained to incoming signals [18, 64]. This phase entrainment was found to be predictive of successful processing. A well-studied model of such processing posits that low frequencies parse incoming information into segments [65] to enable more efficient processing [66]. Our data also show that the amplitude and coherence of cortical oscillations in the theta range are associated with task performance. This frequency range is compatible with possible entrainment by the regular sequences of stimulus presentation used: 500-ms inter-stimulus interval; 2s between consecutive trials. Consistent with previous findings, cortical entrainment to stimulus is expected to vary between trials and participants, and eventually correlate with pitch discrimination performances. Thus, the power of pre-target theta oscillations in right IFG could be a marker of such entrainment to the presented pitch sequences, which we show correlates with performance. However, as the inter-trial intervals were constant, we cannot conclude that entrainment is necessary for the detection of small pitch changes. To determine whether phase entrainment is needed for the detection of small tone deviances, a paradigm with varying inter-trial intervals would be needed as suggested by [32]. That study also pointed out that the detection of near-threshold sounds is independent of the EEG phase, and that the relation between target-detection and delta phase could be due to acausal bandpass filtering. Our analysis of a possible relation between the phase angle did not reveal any preferred phase. At the presentation of the 4^*th*^ tone we found a phase alignment effect across trials within the left hemisphere in the alpha frequency range, which was independent of the detection probability for a pitch change.

Previous studies have found involvement of the right IFG in pitch memory [67–69] and in the processing of targets in musical priming paradigms [70, 71]. Tillmann et al. (2003) in particular, have argued that the inferior frontal cortex is involved in the integration of pitch information over time, which is consistent with the role of IFG in memory tasks. In the present study, we characterized the electrophysiological activity associated with the central role of the right IFG in the fronto-temporal pitch processing pathway. Decreased synchronization in the theta band from 5–8 Hz in the right IFG predicts the correct detection of a near-threshold pitch change. This frequency range corresponds to that found in a previous EEG study on the detection of target sounds in noise, where lower power was also correlated with better performance [14]. Both our study and the study by [14] are thus in agreement with prior work indicating the inhibitory role of the theta band in perception (i.e., [20, 72]). We add to this literature by localizing this preparatory activity at the cortical level, thus extending previous results that have revealed the involvement of theta oscillations in the auditory detection of both pitch oddballs and targets in noise.

To summarize, the current study reveals that decreased synchronization in both IFG predict the successful detection of small pitch changes. These neurophysiological findings strengthen previous results regarding the involvement of fronto-temporal processing in pitch discrimination. More generally, our results provide new insight into the neural encoding preceding an attended auditory target, expanding our knowledge of the time course of auditory processing in the brain.

## Supporting information

S1 Appendix Supporting methods. (PDF)

S1 Table Behavioural data. (PDF)
