# Supplementary material for: Pre-target neural oscillations predict variability in the detection of small pitch changes
Source: PLoS One. 2017 May 18;12(5):e0177836. doi: 10.1371/journal.pone.0177836 (PMC5436812; doi:10.1371/journal.pone.0177836)
Supplement: S1 Appendix — (PDF) [file pone.0177836.s001.pdf]

A Supplementary Methods

A.1 Calculation of the Evoked Response

Denote the subjects with $i = \{1, \ldots, 19\}$ the sources with $j_i = \{1, \ldots, N_{i_j}\}$ where N_{i_j} denotes the subject-specific number of sources, the task type with $k \in \{standard, hard, easy\}$, the number of task repetitions with $l_k = \{1, \ldots, L_k\}$ where L_k is the total number of analyzed trials in each task type, and the time relative to the onset of the fourth tone in milliseconds with $t = \{\ldots, -\frac{1}{2.4}, 0, \frac{1}{2.4}, \ldots\}$. Suppressing the double subscripts, we then compute

$$ERP_{ijkt} = \frac{1}{N_{ik}^{correct}} \sum_{l=1}^{L_k} x_{ijklt} \mathbb{1}_{correct} \, ,$$

where $N_{ik}^{correct}$ is the task- and subject-specific number of correctly detected trials. x_{ijklt} denotes the MEG source time series as well as the 2 EEG sensor time series, and $\mathbb{1}_{correct}$ is an indicator function that takes the value 1 if the tone was correctly detected and 0 otherwise. For this analysis, we used the short trial length $t = [-100, +1000]ms$.

To project the difference between conditions $k = \{hard, easy\}$ on the cortical surface, we computed the difference of the low-pass filtered, absolute values to the standard condition, $ERP_{ijkt}^{\text{diff}} = |ERP_{ijkt}^{\text{filt}}| - |ERP_{ij,standard,t}^{\text{filt}}|$. The data were then standardized with respect to the baseline period, $t = [-100, 0]ms$, by subtracting the mean $\overline{ERP}_{ijk}^{\text{diff}} = \frac{1}{240} \sum_{t=-100}^{0} ERP_{ijk_{diff}t}^{\text{diff}}$ and dividing by the corresponding standard deviation, for $k \in \{hard, easy\}$. Denote the normalized ERP with $ERP_{ijkt}^{\text{diff,norm}}$.

A.2 d' and bias

d' is calculated from each individual subject based on the performance according to the following (Macmillan and Creelman, 2005):

$d' = z(H) - z(F)$

z is the inverse of the normal distribution, H the hit rate and F the false alarm rate. d' was

calculated for the hard versus standard trials. Therefore the hit and false alarm rate were calculated as follows:

$H = \sum_{l=1}^{L_{hard}} \mathbb{1}_{correct}/L_{hard}$

$F = \sum_{l=1}^{L_{standard}} \mathbb{1}_{incorrect}/L_{standard}$

In addition the bias c was determined for the behavioral data:

$c = -\frac{1}{2}[z(H) + z(F)]$.
