# Supplementary material for: Pre-target neural oscillations predict variability in the detection of small pitch changes
Source: PLoS One. 2017 May 18;12(5):e0177836. doi: 10.1371/journal.pone.0177836 (PMC5436812; doi:10.1371/journal.pone.0177836)
Supplement: S1 Table — (PDF) [file pone.0177836.s002.pdf]

A Behavioural Data

Table 1: Behavioural Data
RT- reaction time for correct and incorrect trials per trial type

#	Gender	Age	musical training	accuracy standard	accuracy easy	accuracy hard	d'	c (bias)	RT standard	RT easy	RT hard
1	f	23	0	0.96	1	0.89	2.95	0.23	0.63	0.48	0.69
2	m	29	7	0.97	0.98	0.84	2.86	0.43	0.71	0.65	0.71
3	f	30	13	0.73	1	0.82	1.53	-0.16	0.78	0.56	0.78
4	f	21	0.5	0.93	0.97	0.30	0.96	0.99	0.69	0.61	0.77
5	m	32	0	0.84	0.97	0.70	1.53	0.23	0.68	0.61	0.75
6	m	26	0	0.96	0.98	0.50	1.75	0.87	0.82	0.67	0.93
7	m	23	0	0.98	0.98	0.88	3.21	0.41	0.85	0.78	0.92
8	f	31	2	0.71	0.99	0.97	2.37	-0.64	1.18	0.62	0.87
9	f	35	1	0.98	0.92	0.40	1.79	1.15	0.83	0.80	0.96
10	f	31	4	1	0.99	0.63	3.25	1.31	0.83	0.62	0.94
11	f	41	0	1	0.99	0.19	1.83	1.80	0.60	0.63	0.76
12	m	44	1	0.95	0.99	0.47	1.60	0.88	0.89	0.71	1.08
13	m	24	0	0.99	0.99	0.19	1.61	1.67	0.70	0.61	0.76
14	m	21	4	1	0.99	0.01	0.24	2.60	0.69	0.67	0.73
15	m	24	3	0.95	0.97	0.99	3.89	-0.27	0.88	0.76	0.83
16	f	25	8	1	0.99	0.62	3.23	1.32	0.87	0.92	1.06
17	m	26	3	0.96	0.99	0.65	2.18	0.70	0.82	0.70	0.80
18	m	20	0	1	0.93	0.24	1.99	1.71	0.73	0.76	0.86
19	f	27	0	0.95	0.98	0.42	1.47	0.94	0.75	0.65	0.81
